# Society Meetings

**Published:** 1918-05

**Authors:** 


### SOCIETY MEETINGS

**Brief reports and announcements of meetings of societies of Western New York. and those of general scope, are requested from Secretaries. Copy should be on hand the fifteenth of the month. Full transactions will be published at cost of composition.**

"It was agreed at a meeting held in Paris on November 3rd, 1917, of delegates of the International Surgical Society from Belgium, France, Great Britain, Serbia and the United States of America, that:

1. The International Surgical Society be dissolved after the publication of the Volume of Transactions of the meeting held at New York City, April 14th, 1917. Should any money remain after the publication of the Volume, such money will be divided pro rata among members. Each member of the Austro-German group will receive his share; but the money belonging to members from other nations will be retained and applied to some object of scientific reparation in Belgium.

2. A new Society will be created after the war on a similar basis, to be called the "Inter-Allied Surgical Society." Surgeons of neutral countries may also be elected members."

**Elmira Academy of Medicine.** The regular monthly meeting was held at the Federation Building, Wednesday evening, April 3. Dr. Geo. M. Case, "Relation of Nasal to Eye Disturbances;" Dr. Chas Abbott. E. T. Bush, M. D., President. J. Lee Kinner, M. D., Secretary.

**Rochester Academy of Medicine.** A regular meeting of Section IV was held at the Association rooms, 33 Chestnut St., Tuesday, April 16. Differential Diagnosis and Treatment of Syphilis, (illustrated), Grover W. Wende, M. D., Buffalo. Curtis N. Jameson, M. D., Chairman. Franklin W. Bock, M. D., Secretary.

**Buffalo Academy of Medicine,** Section of Obstetrics and Gynecology. The Regular Meeting of this Section was held in the Academy Rooms, at Orpheus Hall, 423 Franklin St., Wednesday evening, April 17. Abnormal Obstetric Cases and their Treatment, Dr. James W. Markoe, New York City. H. K. DeGroat, M. D., Chairman. E. A. D. Clarke, M. D., Secretary.

The regular meeting of **Genesee County Medical Society** was held at the Holland Club, Batavia, at 8.30 p. m. April 10. Program: Dynamics of Hernia, Dr. Harry Trick of Buffalo. Discussed by Dr. W. D. Johnson. Venereal Diseases, Dr. Edward Clark of Buffalo. Dr. Albert T. Lytle, president of 8th district branch was also present. Dr. Edgar Bieber of Byron, lieut. M. R. C., left for Base Hospital, Camp Dix, April 16. Edith F. Ryan, Secretary.

